# Musculoskeletal injuries and absenteeism among healthcare professionals—ICD-10 characterization

**DOI:** 10.1371/journal.pone.0207837

**Published:** 2018-12-14

**Authors:** João Amaro, João Magalhães, Margarida Leite, Beatriz Aguiar, Paula Ponte, Joana Barrocas, Pedro Norton

**Affiliations:** 1 Department of Occupational Health, Centro Hospitalar Universitário São João, Porto, Portugal; 2 EPIUnit – Instituto de Saúde Pública, Universidade do Porto, Porto, Portugal; 3 USF Espaço Saúde, ACeS Porto Ocidental, Porto, Portugal; 4 USF Brás Oleiro, ACeS Porto Ocidental, Porto, Portugal; 5 USF Prelada, ACeS Porto Ocidental, Porto, Portugal; 6 USF Maresia, ULS Matosinhos, Leça da Palmeira, Portugal; Larry Katz School of Medicine, Temple University, UNITED STATES

## Abstract

**Introduction:**

Healthcare workers account for 10% of the EU’s total workforce, with a significant proportion of those employed in hospitals. Musculoskeletal injuries are the predominant group of injuries in healthcare professionals due to the physical demands of their work, such as the mobilization and positioning of the dependent patients. The management of this type of problem should take into account direct and indirect costs, such as periods of incapacity for work due to illness, hiring and training of new employees during periods of absence, reduced levels of productivity and the effects on production and quality of work.

**Objectives:**

1—Characterization of injuries resulting from occupational accidents in hospital workers according to the International Classification of Diseases ICD-10; 2—Identification of the predictive factors of absenteeism duration due to temporary work incapacity in workplace accidents.

**Methods:**

A retrospective observational study was conducted based on the analysis of 1621 cases of work-related accidents of employees of Centro Hospitalar São João from January 2011 to December 2014.

An ICD-10 classification code was associated with each of the accident cases, based on pre-established criteria for classification of the specific diagnoses of musculoskeletal injuries. The duration of temporary work incapacity was compared between the categories of sociodemographic variables, among six categories of ICD-10 primary diagnosis (reclassification), and between the two major chapters of ICD-10 classification—chapter XIX (direct trauma) and chapter XIII (indirect trauma—strain injuries). The sociodemographic predictors of the occurrence of strain injuries were determined by logistic regression. A multinomial logistic regression analysis was conducted with selection of duration of work incapacity as the dependent variable.

**Results:**

A total of 824 cases of musculoskeletal injuries occurred on hospital premises during the study period, which corresponded to a total of 22159 lost workdays in the context of temporary work incapacity due to work injury. According to the ICD-10 reclassification, the three most frequent diagnostic groups were direct lower limb trauma (n = 230, 27.9%), spinal strain injuries (n = 194, 23.5%) and direct upper limb trauma (n = 174, 21.1%). Significant differences were observed in temporary work incapacity duration among the ICD-10 diagnostic categories: spinal strain injuries were the diagnostic group associated with longer duration of temporary work incapacity, with a median = 14.0 (25-75th percentile: 6.0–35.0). The only variable that demonstrated to be significantly predictive of temporary work incapacity less than or greater than 20 days was the ICD-10 diagnostic group. The regression results revealed a 5-fold increase in risk in the case of spinal strain injuries for temporary work incapacity durations of less than or greater than 20 days (OR = 5.58 and OR = 5.89 respectively).

**Conclusions:**

The study findings support the benefits of the characterization of workplace injuries by medical diagnostic groups, namely in the interpretation of the sequelae of the accidents and the medical contextualization of the accidents. Association of ICD-10characterization can improve the analysis of workplace accidents at an institutional level, and promote the implementation of preventive measures and control of absenteeism.

## Introduction

According to the current portuguese legislation, a work accident is one that “happens on site and during working time and produces a direct or indirect body injury, functional disorder or illness resulting in reduction of working or earning capacity, or death" [1]. Work accidents entail several negative consequences, for the employer and the employee, either for the possible compromise of both physical and mental health of the latter, as for the negative impact on labor dynamics, organizational productivity and the high associated costs. In fact, the human cost of this daily adversity is vast with estimates of 1 to 3% of GDP in some european countries [2–4].

In recent years, a growing concern for safety at work by the government and employers has been observed, which has resulted in the development of policies regulating employee safety measures, nationwide and worldwide. As a result, european data show a decline of 17.4% work accidents resulting in more than three days of absence between 1995 and 2005, and a reduction of 35.6% regarding fatal accidents over the same period [5]. In Portugal, the number of accidents has also decreased, with 193 611 reported cases in 2012 (175 of them fatal), compared to 233 217 (348 fatal) in 1985 [6].

The employees of the healthcare sector account for 10% of the total workforce of the European Union [7], a significant proportion of which are employed in hospitals. While it is known that the manufacturing and construction sectors are theoretically the ones associated with greater danger to employees, the risks to which healthcare employees are exposed cannot be ignored, with some national occupational health reports showing a global incidence of workplace injuries in the healthcare sector higher than in other professional sectors such as those mentioned [8]. According to the Portuguese Central Administration of Health Services (ACSS), in 2013, for every 1000 employees of the National Health Service (NHS), 52.93 were victims of a work accident, 75% corresponding to nursing assistants and nurses [9].

Musculoskeletal injuries (MSIs) related to work represent a heterogeneous group of clinical conditions involving the musculoskeletal system that occur by exposure to various risk factors at the workplace [10], and are the predominant lesion of healthcare workers due to the physical demands of their type of work, such as patient handling or push-pull tasks. The most affected regions of the body are the lower back, neck and shoulders and hand/wrist [11–13].

In the management of these disorders one must consider not only direct costs (insurance, compensation, medical and administrative costs) but also indirect costs such as sick leave periods, hiring and training of new employees in absence periods, reduced productivity levels and effects on production and quality of work [2]–some authors argue that the direct costs represent a small proportion of the global economic burden of workplace MSIs [14]. A recent Portuguese national report indicated that 5.161.343 work days were lost due to workplace accidents in 2013 and that 21,2% of these episodes led to an absence period superior to 30 days [15].

European regulation on health and safety at work [16] sets out obligations to supply statistics on accidents at work; this harmonized data and information to be provided on accidents at work cover several subjects such as characteristics of the injured person and characteristics of the injury, including severity (days lost). Although useful for national/regional harmonization of work accident statistics, the recommended categories used in this context for injury characterization lack specific and reliable characterization of the physical consequences of the injury, dedicating a much more extensive detail to the characterization of the injury mechanism. As an example, according to the recommendations of the European Statistics on Accidents at Work [17], the Type of Injury (the variable which is closest to what one can assume as diagnostic) includes generalist groups of terms such as "Fractures", "Dislocations, sprains and strains", "Concussions and internal injuries" or "Burns, scalds, freezing" (among others). In our experience these terms do not provide an accurate medical diagnostic characterization of the injury that could be relevant for the analysis of work accidents at an individual or institutional level (e.g.: specific injury types that occur in a given department or professional group) or prediction of work absence (according to medical diagnostic of the injury). To our knowledge this subject has not yet been addressed in previous research.

## Objectives

1—Characterization of injuries resulting from occupational accidents in hospital workers according to the International Classification of Diseases ICD-10; 2—Identification of the predictive factors of absenteeism duration due to temporary work incapacity in workplace accidents.

## Materials and methods

The present study is a retrospective study on 1621 cases of work accidents suffered by hospital employees occurring from January 2011 to December 2014; a specific database on work accidents was organized from January 2011. The data was collected from clinical records of the hospital’s occupational health department. The sociodemographic characteristics of employees were obtained from the Hospital’s Human Resources department.

Information was collected on: Patient age, Gender, Professional group, Seniority in the institution, Level of education, Type of injury (biological/chemical vs. musculoskeletal vs. others), Type of work incapacity (none, partial or absolute), Duration of work absence—absenteeism due to injury and Primary diagnosis according to International Classification of Diseases and related health problems (ICD-10—version 2015). For this study, only cases with absolute work incapacity immediately following the injury were considered as periods of work absence.

Cases of accidents with biological risk (such as needle injuries), injuries with chemical substances, superficial skin injuries and accidents “in itinere” were excluded from the descriptive analysis and regression. Accidents “in itinere” were excluded from the analysis due to the particular characteristics of the injury mechanisms.

### ICD-10 classification of work injuries

MSIs were classified according to the ICD-10 (version 2015), as seen in Table 1. Four family medicine residents conducted the data collection and episode classification. Prior to data collection, a consensus regarding ICD-10 diagnostic classification of musculoskeletal injuries was achieved, in order to improve validity and inter-observer reliability; the consensus criteria was established by a panel of three medical specialists: family medicine, occupational health and physical medicine and rehabilitation. The consensus for ICD-10 classification included criteria such as: classification of musculoskeletal injuries involving direct trauma under chapter XIX; classification of MSIs involving injuries with strain/sprain mechanism or indirect trauma (e.g.: lumbago or shoulder pain from load handling) under chapter XIII; primary classification of direct injuries (chapter XIX) according to anatomical location; hand/wrist injuries and ankle/foot direct injuries were subdivided in superficial and deep categories (e.g.: wrist contusion vs. wrist fracture). After the classification of each injury, the primary diagnosis was reclassified into six main categories for analytic purposes: strain injuries of vertebral column; strain injuries of upper and lower limbs and arthropathies; direct head trauma; direct trauma to vertebral column, thorax, abdomen or pelvis and polytrauma; direct upper limb trauma; direct lower limb trauma.

**Table 1:** Classification criteria according to ICD-10.

Variable number	ICD-10 main chapter	ICD-10 coding	Specific diagnoses	Reclassification
1	XIII	M53.1, M54.2	Dorsopathies—cervical spine	Spinal strain injuries
2	XIII	M54.4, M54.5	Dorsopathies—lumbar spine	
3	XIII	M54.6, M54.8	Other dorsopathies	
4	XIII	M62.1, M62.4	Other soft tissue injuries—muscular injuries	Strain injuries of upper and lower limbs and arthropaties
5	XIII	M75	Soft tissue injuries—tendinous or synovial injuries—shoulder injuries	
6	XIII	M77	Soft tissue injuries—tendinous or synovial injuries—other entesopathies of the UL or LL	
7	XIII	M23, M25.5, M99.5	Arthropathies	
8	XIX	S00.1, S00.3, S00.5, S00.8, S00.9, S01.0, S01.1, S01.2, S01.5, S02.1, S02.2, S02.5, S05.1, T14.0	Head injuries (excl. TBI)	Direct head trauma
9	XIX	S06.0	Traumatic Brain Injury	
10	XIX	S10.1, S10.9, S12.2, S19.9	Cervical injuries	Direct trauma to vertebral column, thorax, abdomen or pelvis; polytrauma
11	XIX	S20.0, S20.2, S22.3, T09.0	Thoracic injuries	
12	XIX	S30.0, S30.2, S32.1, S32.2, S32.3, S33.5, S39.9	Abdominal, lumbar spine and pelvic injuries	
13	XIX	T00.8, T00.9, T11.0	Multiple injuries; polytrauma	
14	XIX	S40.0, S42.3, S42.4, T14.6	Shoulder and arm injuries	Direct upper limb trauma
15	XIX	S50.0, S50.1, S50.8, S52.0, S52.1, S52.5, S52.9, S53.1, S53.4, S59.1, S59.9	Elbow and forearm injuries	
16	XIX	S60.0, S60.1, S60.2, S60.8, S61.0, S61.8, T14.0	Wrist and hand injuries—superficial	
17	XIX	M20.0, S62.0, S62.3, S62.5, S62.6, S62.8, S63.4, S63.6, S63.7	Wrist and hand injuries—bone and ligament injuries	
18	XIX	S70.0, S70.1	Hip and thigh injuries	Direct lower limb trauma
19	XIX	S80.0, S80.1, S82.0, S82.4, S82.6, S82.7	Knee and leg injuries—direct trauma	
20	XIX	M24.2, S83.2, S86.0, S86.1, T13.5	Articular injuries of the knee ligament rupture of knee/leg	
21	XIX	S90.0, S90.1, S90.2, 90.3, S90.8, S90.9, S91.0	Ankle and foot injuries—superficial	
22	XIX	S92.3, S92.4, S92.5, S93.4, S93.6	Ankle and foot injuries—bone and ligament injuries	

### Identification of predictive factors for absenteeism—Descriptive analysis

The possible predictive factors for absenteeism due to injury included in this study can be divided into two major groups: sociodemographic variables (gender, age, years of hospital work, level of education, professional group) and ICD-10 diagnosis; duration of work absence was defined as the dependent variable.

Professional groups were classified in six categories according to the type of work: medical staff (e.g.: medical specialists or residents), registered nursing personnel, nursing assistants, healthcare technicians (e.g.: radiology technicians, laboratory technicians, physical therapists), administrative assistants and other professional groups (e.g.: hospital managers, IT technicians). Level of education was classified into three categories: university graduate, high school degree or professional/technical course (12 years of education) and under 9 years of education. For descriptive purposes both age and years of hospital work were coded into five categories. The six variable categories derived from ICD-10 reclassification were used for statistical analysis.

Prior to the conduction of the multinomial logistic regression model, for analysis purposes, duration of work absence was compared across all independent variable categories; duration of work absence was also compared between the two major sections of MS injury—direct trauma (chapter XIX) and indirect trauma (chapter XIII). Prior to the conduction of the regression model an analysis on the association between the several independent variables was also conducted in the model for assessment of collinearity.

### Multinomial logistic regression

Independent variables were modified for inclusion in the regression model. ICD-10 classification categories (after reclassification) were grouped into five final categories, with two of the former (“Direct head trauma” and “Direct trauma to vertebral column, thorax, abdomen or pelvis/polytrauma” being grouped into one category—“Other injuries due to direct trauma/polytrauma”. Professional group and age were grouped in three categories each. Years of hospital work were grouped in two categories: [less than 10 years; superior to 10 years]; level of education was grouped in two categories: [Primary school, high-school or technical course, university education].

A multinomial logistic regression with duration of work absence as the dependent variable was conducted. ICD-10. The reference categories for the final model independent variables were female (variable gender), more than 50 years (variable age), other professional groups (variable professional group) and other direct injuries / polytrauma (variable ICD-10 classification). Years of hospital work and level of education were excluded from the final model. The dependent variable was coded into three categories—no absence from work, work absence lasting less than 20 days, work absence lasting more than 20 days; no work absence was defined as the reference category.

### Statistical tests

Variables with asymmetrical distribution were described with median values and percentile values at P25 and P75. Non-parametric statistics was used for comparison between distributions of work absence duration between categories—Mann-Whitney test for two samples and Kruskal-Wallis test for multiple samples—since the frequency distribution of this variable was found to predominantly right-skewed. Analysis of statistically significant differences between frequency distribution of categorical variables was conducted with Pearson Chi-square; Spearman coefficient was used for correlation analysis between continuous variables (age and years of hospital work). Statistical analysis was performed with SPSS (version 24.0); 0.05 was defined as the limit for statistical significance.

### Ethical considerations

This study was approved by the Ethics Committee of the Centro Hospitalar São João. To ensure the confidentiality of all the collected and analyzed data, an encoding of individuals in a separate database was conducted, allowing subsequent access to information and maintaining the confidentiality of the data.

## Results

A total of 824 cases of musculoskeletal injury was obtained after exclusion of *in itinere* accidents, biological risk injuries (e.g. needle stick injuries) and superficial skin injuries, which corresponded to a total of 22.159 lost work days due to injury. Most of the MSIs occurred in female workers (n = 673, 81.7%), with ages between 30–40 years (n = 271, 32.9%), between 5–10 years of hospital work (n = 233, 28.3%), with higher education (n = 363, 44.1%); nursing assistants were the most affected professional group (n = 426, 51.7%).

### ICD-10 classification of work injuries

The most frequent diagnoses are displayed in descendent order of frequency in Table 2—prior to reclassification. Dorsopathies—lumbar spine, ankle and foot injuries and wrist and hand injuries are the three most frequent diagnosis (17.5%, 11.9% and 11.3% respectively); shoulder injuries with indirect trauma are also shown to be relevant in terms of frequency (n = 54, 6.6%). Regarding duration of work absence it is also possible to ascertain that besides some direct trauma injuries (wrist/hand, ankle/foot and shoulder), lumbar strain, shoulder strain and cervical strain injuries are responsible for prolonged median periods of work absence (14, 14 and 13 median days of work absence, respectively).

**Table 2:** Frequency distribution of ICD-10 diagnosis; median duration of work absenteeism and cumulative lost workdays per ICD-10 diagnosis.

ICD-10 Diagnosis	Freq. (perc.)	Median duration of work absence (days)	Lost work days, (n)
Dorsopathies—lumbar spine	144 (17.5)	14	4537
Ankle and foot injuries—bone and ligament injuries	98 (11.9)	14	3154
Wrist and hand injuries—superficial	93 (11.3)	4	1557
Soft tissue injuries—tendinous or synovial injuries—shoulder injuries	54 (6.6)	14	2099
Knee and leg injuries—direct trauma	47 (5.7)	13	1707
Soft tissue injuries—tendinous or synovial injuries—other entesopathies of the UL or LL	40 (4.9)	4	708
Dorsopathies—cervical spine	39 (4.7)	13	851
Articular injuries of the knee; ligament rupture of knee/leg	38 (4.6)	10	1370
Ankle and foot injuries—superficial	37 (4.5)	7	544
Shoulder and arm injuries (direct trauma)	32 (3.9)	17	1284
Traumatic Brain Injury	27 (3.3)	5	333
Elbow and forearm injuries (direct trauma)	27 (3.3)	6	823
Thoracic injuries	24 (2.9)	0	252
Head injuries (excl. TBI)	22 (2.7)	0	369
Abdominal, lumbar spine and pelvic injuries	22 (2.7)	6,5	687
Wrist and hand injuries—bone and ligament injuries	22 (2.7)	20,5	777
Other soft tissue injuries—muscular injuries	18 (2.2)	15,5	403
Other dorsopathies	11 (1.3)	12	150
Arthropathies	10 (1.2)	7,5	240
Hip and thigh injuries	10 (1.2)	8	188
Cervical injuries	6 (0.7)	7,5	90
Multiple injuries; polytrauma	3 (0.4)	0	36
**Total**	824 (100)	9	22159

### Identification of predictive factors for absenteeism—Descriptive analysis

Distribution of work absence duration was compared between SD categories (Table 3) and ICD-10 diagnosis after reclassification (Table 4). There were no statistically significant differences in the duration of work absence among sociodemographic categories—the largest differences were found between age groups, with longer work absence due to injury in more advanced ages (from 40 years), but without reaching statistical significance (p = 0.069). On the other hand, significant differences in work absence duration were observed among the ICD-10 diagnostic categories (after reclassification): spinal strain injuries were the diagnostic group associated with longer duration of work absence with med. = 14.0 (25^th^-75^th^ percentile: 6.0–35.0), compared to other diagnostic groups such as direct upper limb trauma, with med. = 7.0 (0.0–27.5), direct lower limb trauma with med. = 10.0 (0.0–37.5), or strain injuries of upper and lower limbs with med. = 10.0 (0.0–36.5). Overall, strain injuries were associated with longer duration of work absence in comparison to direct trauma (med. = 13.0 vs. med. = 7.0, p = 0.001).

**Table 3:** Frequency distribution and cumulative lost workdays per sociodemographic category; comparison of median duration of work absence across sociodemographic categories.

Sociodemographic variables	Freq. (perc.)	Lost work days, n (perc.)	Median duration of work absence (days)	p
**Gender, n (%)**				0.378
Male	151 (18.3)	4019 (18.1)	17.0	
Female	673 (81.7)	18140 (81.9)	21.0	
**Age group, n (%)**				0.069
Less than 30 years	116 (14.1)	2027 (9.2)	14.5	
30–40	271 (32.9)	6146 (27.7)	14.5	
40–50	221 (26.8)	6520 (29.4)	26.5	
50–60	192 (23.3)	6157 (27.8)	22.5	
Over 60 years	24 (2.9)	1309 (5.9)	33.0	
**Seniority in the institution, n (%)**				0.582
Less than 5 years	177 (21.5)	3847 (17.4)	18.0	
5–10	233 (28.3)	5934 (26.8)	17.0	
10–15	151 (18.3)	4880 (22.0)	23.0	
15–20	122 (14.8)	3848 (17.4)	22.0	
Over 20 years	141 (17.1)	3650 (16.5)	22.0	
**Level of education, n (%)**				0.135
Primary school (up to 9 years)	330 (40.0)	10800 (48.7)	21.0	
High school / technical course	131 (15.9)	3085 (13.9)	20.5	
University graduate	363 (44.1)	8274 (37.3)	17.0	
**Professional group, n (%)**				0.557
Nursing assistants	426 (51.7)	12897 (58.2)	20.5	
Registered nursing personnel	284 (34.5)	5746 (25.9)	16.0	
Healthcare technicians and other professional groups	29 (3.5)	1246 (5.6)	23.0	
Administrative assistants	44 (5.3)	1104 (5.0)	24.0	
Medical staff	41 (5.0)	1166 (5.3)	28.0	

**Table 4:** Frequency distribution and cumulative lost workdays per ICD-10 category (after reclassification); comparison of median duration of work absence across ICD-10 categories.

ICD-10 reclassification	Freq. (perc.)	Lost work days, n (perc.)	Median duration of work absence (days)	p
**Diagnostic group**				<0.001
Spinal strain injuries	194 (23.5)	5538 (25.0)	14.0	
Strain injuries of upper and lower limbs and arthropaties	122 (14.8)	3450 (15.6)	10.0	
Direct head trauma	49 (5.9)	702 (3.2)	0.0	
Direct trauma to vertebral column, thorax, abdomen or pelvis; polytrauma	55 (6.7)	1065 (4.8)	3.0	
Direct upper limb trauma	174 (21.1)	4441 (20.0)	7.0	
Direct lower limb trauma	230 (27.9)	6963 (31.4)	10.0	
**ICD-10 Chapter**				0.001
Strain injuries (chap. XIII)	316 (38.3)	8988 (40.6)	13.0	
Direct trauma (chap. XIX)	508 (61.7)	13171 (59.4)	7.0	

For assessment of collinearity, prior to the conduction of the regression model, the analysis on the association between sociodemographic variables and diagnostic groups showed significant differences in the distribution of the ICD-10 diagnostic groups across age categories, seniority in the institution and professional groups. There was a greater proportion of spinal strain injuries amongst younger workers (up to 35 years) compared to workers older than 50 years (n = 88, 32.7% vs. n = 32, 13.8%, respectively). Similar differences were recorded in the same age groups in the case of strain injuries of upper and lower limbs (n = 49, 18.2% vs. n = 21, 9.1% respectively). On the other hand, lesions with direct lower limb trauma were more frequent in older workers (n = 58, 21.6% vs. n = 89, 38.4%, respectively, for the same age groups).

Spinal strain injuries were more prevalent in nursing assistant and nurses, in comparison with other professional groups (n = 103, 24.2%, n = 81, 28.5% and n = 10, 8.8%, respectively). On the other hand, direct lower limb trauma injuries were more prevalent in other professional groups (n = 115, 27.0%, n = 67, 23.6% and n = 48, 42.1%). As in the age groups, there were also significant differences in the type of injury according to seniority in the institution. There were no statistically significant differences in the distribution of the ICD-10 diagnostic groups across the variables of gender and level of education.

### Multinomial logistic regression

**Fig 1:**
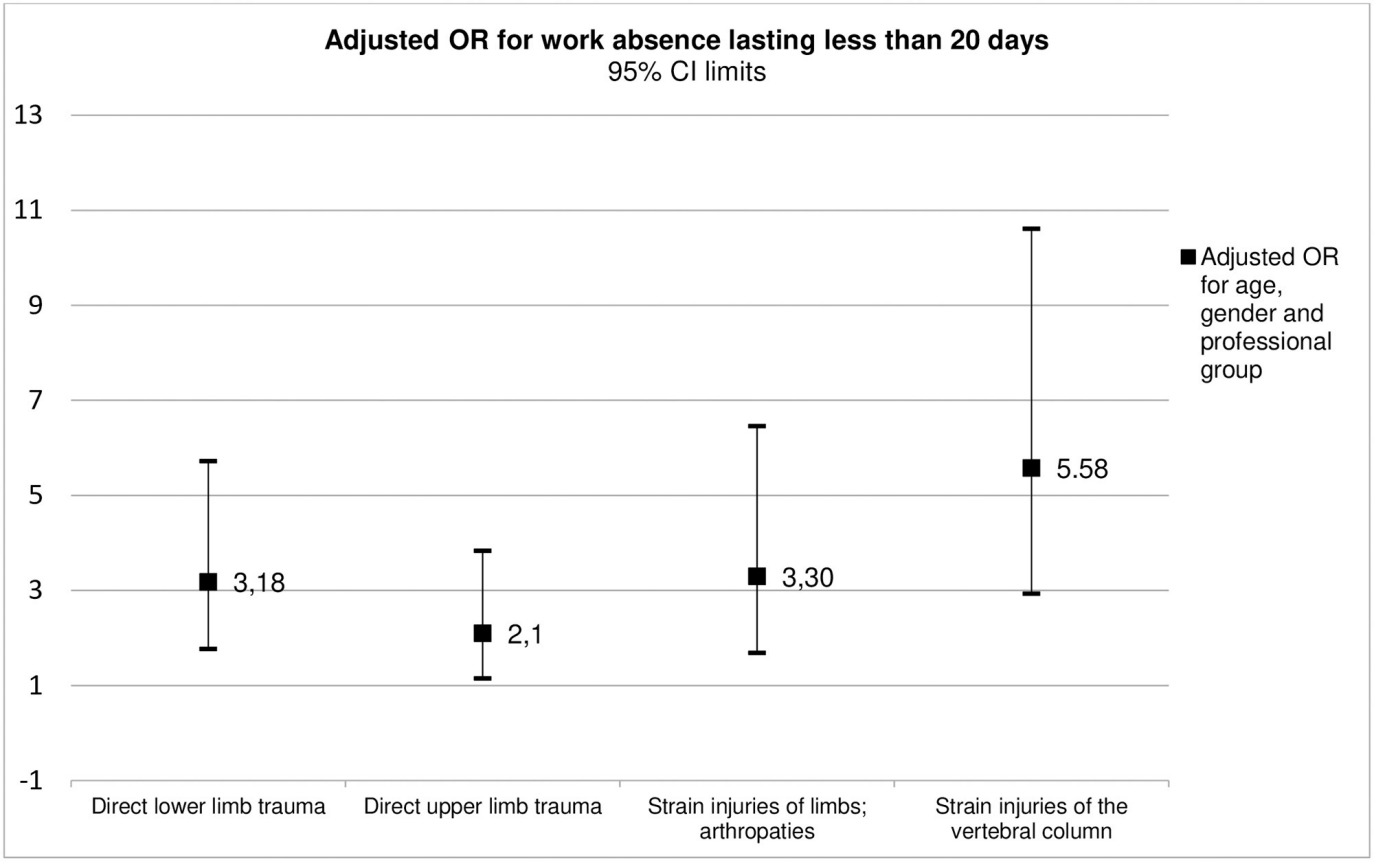
Adjusted OR of the ICD-10 diagnostic groups for work absence lasting less than 20 days.

**Fig 2:**
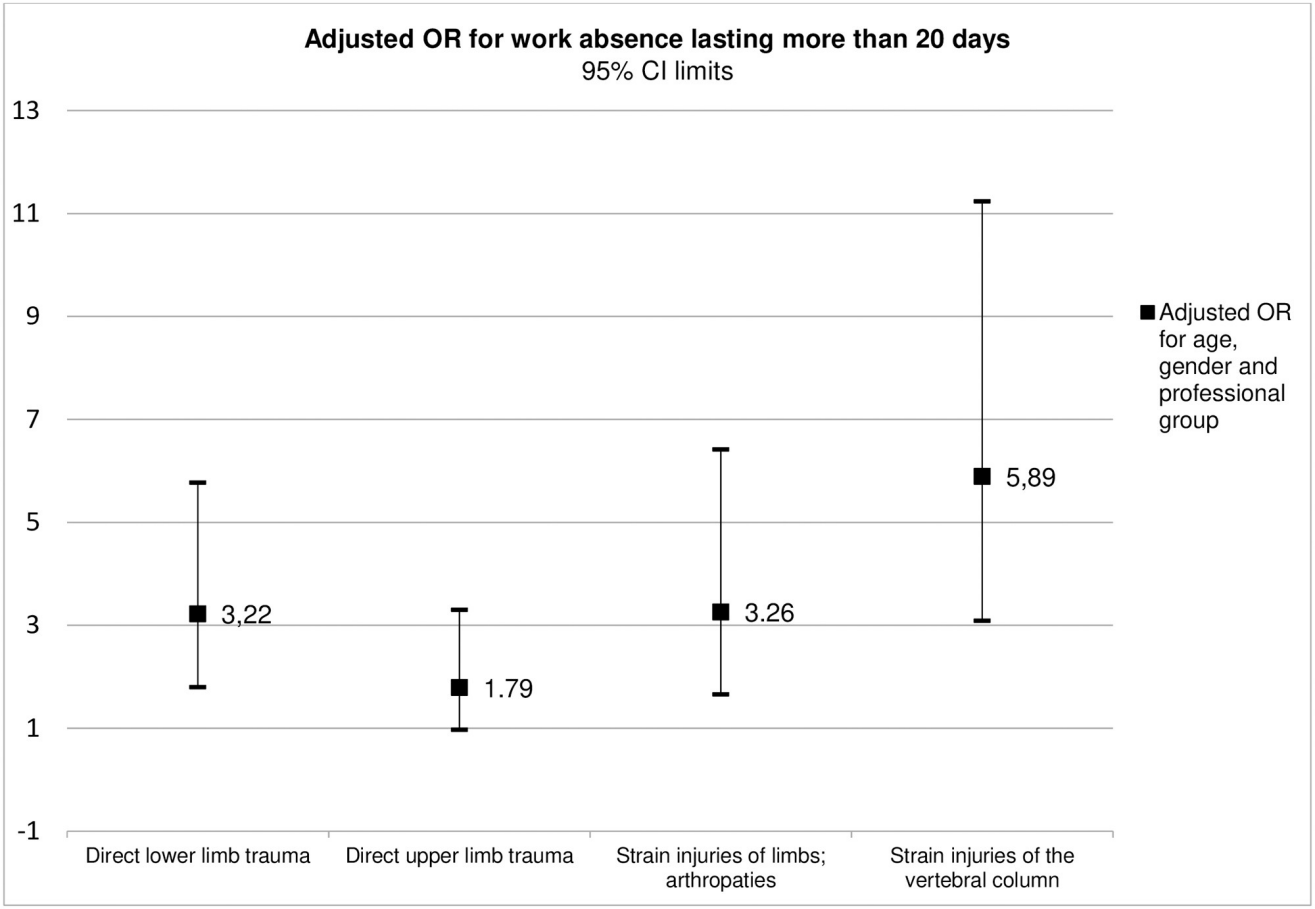
Adjusted OR of the ICD-10 diagnostic groups for work absence lasting more than 20 days.

The variables seniority in the institution and education level were excluded from the final model due to associations with other SD categories and because they did not significantly alter the adjustment of the model. The only variable demonstrated to be a significant predictor of work absence lasting less or more than 20 days was the ICD-10 diagnostic group—Figs 1 and 2. Spinal strain injury was the diagnostic category associated with the most increased risk of work absence, with similar risk values for work absence lasting less or more than 20 days: OR = 5.58 (CI95% 2.93–10.61) and OR = 5.89 (CI95% 3.09–11.23), respectively.

## Discussion

Musculoskeletal injuries occurring in the context of work-related accidents cover the most varied diagnoses, ranging from polytrauma with fractures in multiple joint regions, brain or medullar trauma with severe neurological sequelae, to exertional lower back pain or overload injury of the upper or lower limbs (e.g.: shoulder tendinitis). In general acute MSIs are mostly associated with direct trauma injuries; however our results indicate a high prevalence of strain (indirect) injuries, when compared with direct trauma: lumbar strain injuries and shoulder injuries are the most and fourth most frequent ICD-10 diagnosis (17.5% and 6.6% respectively). These results highlight the profile of work accidents in the healthcare sector [8,12] with higher incidences of strain injuries. The association of a more specific diagnostic characterization of these injuries contributed to a more comprehensive understanding of the work injury phenomenon in the study population.

In this study, the distribution of MSI frequency and cumulative number of lost work days by sociodemographic category partially reflects the sociodemographic distribution profile of the hospital population—the majority of cases occurring in female workers (81.7%), aged between 30–40 years (32.9%), seniority in the institution between 5–10 years (28.3%). It is not possible to draw adequate conclusions about the influence of sociodemographic determinants on injury frequency since the study population included only injured workers and did not exclude cases of workers with multiple accidents. A previous study on the sociodemographic determinants MSI in this hospital population was conducted [18] in which nursing assistants presented a 12-fold higher risk of work-related musculoskeletal injury and the in age groups with the highest risk of MSI were between 35 and 49 years old.

In what concerns the analysis of work absenteeism duration, no relevant differences were observed between sociodemographic categories, except for the differences observed between the age groups (with an increase in work absence duration from 40 years of age). This finding is in sharp contrast with the differences observed between ICD-10 diagnostic groups, where spinal strain injuries stand out with a median of 14 days of work absence, followed by strain injuries of the upper and lower limbs and direct lower limb trauma (both with 10 day median). The impact of strain injuries on work absence is further supported by the significant differences found between the duration of work absence of the injuries classified in chapters XIII (strain injuries—indirect trauma) and XIX (direct trauma) of the ICD-10: although direct trauma injuries were more frequent (61.7%), which corresponded to a superior proportion of total lost work days (n = 13.171, 59.4%), median duration of work absence per case was significantly higher in strain injuries (med. = 13.0 vs. med. = 7.0).

The differences in duration of work absence according to the diagnostic groups reflect the nature of occupational injuries in healthcare professionals, in which strain injuries are predominant, with muscular and ligament structures being overloaded by indirect trauma [4, 12, 19, 20]. These lesions, which particularly affect the lumbar spine, cervical spine and shoulders [12, 21, 22] are associated with the positioning of dependent patients in bed, as well as other tasks such as mobilization of patients in manual lift, vertical transfer or hygiene care. These observations should suggest the implementation of measures that address this phenomenon, both preventive as well in a work incapacity context, with specifically developed return to work strategies. In our experience these injuries are also frequently neglected and under-treated by insurance companies.

The analysis on the association between sociodemographic variables and diagnostic groups revealed that strain injuries were particularly incident in younger (up to 35 years) and less senior workers; on the other hand, lesions with direct lower limb trauma were more frequent in older workers. This observation could suggest an education and information deficit on the matter of ergonomics in health care settings in early stages of their professional career, particularly on the occupational risk related to mobilization and positioning of dependent patients. However, these findings may be biased by the fact that older workers are more conditioned and limited in patient handling tasks, possibly by chronic accumulation of occupational and natural injuries, and are placed in settings that do not involve this type of tasks.

The ICD-10 diagnosis was the only variable that was shown to be a significant predictor of the duration of work absence. In spinal strain injuries there was a 5-fold higher risk of work absence, both of absenteeism durations less or greater than 20 days (OR = 5.58 and OR = 5.89 respectively). In a similar way, upper and lower limb strain injuries lesions and direct lower limb trauma were associated with a 3-fold higher risk of work absence, both of absenteeism durations less or greater than 20 days (OR = 3.30 and OR = 3.26 respectively). The fact that this risk is similar across categories of duration of work absence suggests that this predictive effect is not related in a linear manner to the actual duration of work absence.

Limitations of the study include the fact that the study population was only composed by injured workers, lack of test of inter-rater reliability of ICD-10 classification, limited study sample for conduction of the regression model and reduced number of independent variables.

## Conclusions

Occupational health professionals should be increasingly aware of the diagnostic specificities and injury mechanism of work injuries in healthcare workers. This knowledge can be helpful in the design of proper prevention strategies of work injuries, in the management of work absence, and in the return to work period—partial activity restriction and task adaptation. The study findings can support the advantages of the improvement of medical diagnostic characterization of occupational injury episodes, in a complementary way to the standard safety and health characterization of work accidents. Improving diagnostic characterization of occupational lesions can have beneficial consequences on the individual workup of MSIs, in understanding the phenomenon of occupational injury at an institutional level and on assisting the implementation of preventive measures of work injury and absenteeism.

The incidence of strain injuries in younger healthcare workers as well as its impact in work absenteeism should support the promotion of preventive and injury management strategies, in order to achieve a global reduction of injury frequency and absenteeism. The influence of spinal strain injuries on work absence at an individual and institutional level may indicate the need to reinforce preventive measures such as education of workers on the etiology of work injuries in this setting and ergonomic principles in patient handling tasks.

## Supporting information

S1 File Minimal data set. (XLSX)
